# Biochemical Modulation of Fe_2_O_3_/CuFe_2_O_4_ Nanocomposite via Two‐Stage Calcination: Antimicrobial Efficacy and In Vivo Toxicological Profiling

**DOI:** 10.1002/cbdv.71717

**Published:** 2026-09-27

**Authors:** Riyadh Zainadin Mawlood, Chawan Hazhar Razaq, Karukh Ali Babakr, Ibrahim Nazem Qader, Sleman Yousif Omar, Asmaa Sayed Ahmed, Zana Hassan Ibrahim, Mustafa Ersin Pekdemir

**Affiliations:** ^1^ Chemistry Department College of Science University of Raparin Sulaymaniyah Iraq; ^2^ Department of Biology College of Science University of Raparin Sulaymaniyah Iraq; ^3^ Department of Physics College of Science University of Raparin Sulaymaniyah Iraq; ^4^ Department of Medical Laboratory Science College of Science University of Raparin Sulaymaniyah Iraq; ^5^ Polymer and Biomaterials Research Laboratory Department of Chemistry Faculty of Science Fırat University Elazığ Türkiye

**Keywords:** CuFe_2_O_4_, Fe_2_O_3_, heterostructure nanocomposite, in vivo toxicological, microbial activity

## Abstract

Fe_2_O_3_/CuFe_2_O_4_ nanocomposite fabricated by a two‐stage calcination process. X‐ray diffraction established that α‐Fe_2_O_3_ is the predominant phase, accompanied by little CuFe_2_O_4_ spinel, with no extraneous impurity phases identified. In vitro bioassays exhibited robust broad‐spectrum antibacterial efficacy of the nanocomposites. Agar‐diffusion assays demonstrated that sample BB2 (300°C and 300°C) yielded the most substantial inhibitory zones against *Salmonella typhimurium* (22.0 ± 0.5 mm) and *Staphylococcus aureus* (22.0 ± 0.3 mm), far surpassing antibiotic controls (11–14 mm). Significantly, only BB3 and BB4 demonstrated inhibitory effects on *Candida albicans* (zones 14.0–18.0 mm). MIC/MBC assays validated these trends: BB4 demonstrated the lowest MIC/MBC (25/50 µg/mL) against both *S. typhimurium* and *S. aureus*, whereas BB2/3/4 inhibited *C. albicans* at 25/50 µg/mL. All samples significantly impaired biofilm formation (50%–82% decrease), with BB2 achieving around 80% suppression across all pathogens. The results demonstrate that the two‐stage calcined Fe–Cu mixed oxide successfully eliminates both Gram‐positive and Gram‐negative bacteria, as well as fungi. In vivo toxicological assessment revealed no notable deleterious effects on hepatic function, indicating preliminary hepatic tolerability. The optimized Fe_2_O_3_/CuFe_2_O_4_ nanocomposite has significant multi‐modal antibacterial effectiveness and minimal acute toxicity, underscoring its potential for innovative infection‐control applications.

## Graphical abstract

Fe_2_O_3_/CuFe_2_O_4_ nanocomposites with wide‐spectrum antimicrobial activity were fabricated using a two‐step calcination method. The nanocomposites strongly inhibit both Gram‐positive and Gram‐negative bacteria, suppress biofilm formation, and show antifungal activity. The preliminary in vivo assessment shows hepatic tolerability of the nanocomposites. Therefore, the nanocomposites are potential candidates for infection‐control applications.

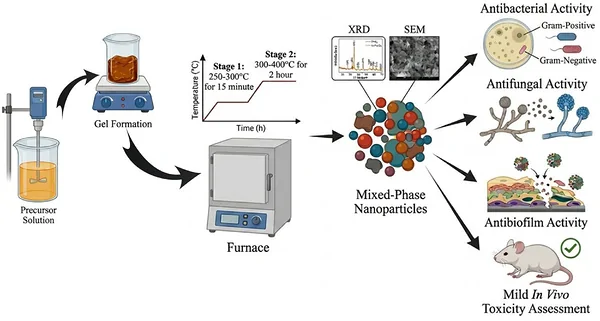

## 1 Introduction

Metal oxide nanocomposites have emerged as versatile platforms for advanced functional materials and biomedical applications. By combining two or more oxide phases at the nanoscale, one can harness synergistic effects that enhance optical, electrical, catalytic, and antimicrobial properties beyond those of the individual components [1, 2]. For example, copper oxide (CuO) and iron oxide (Fe_2_O_3_) are inexpensive, non‐toxic semiconductors with complementary properties: CuO is a p‐type semiconductor with a narrow bandgap (∼1.2 eV) and known biocidal activity [3], while α‐Fe_2_O_3_ (hematite) is an n‐type semiconductor (Eg ≈ 2.1 eV) that is chemically stable and abundant [4]. Individually, iron oxide nanoparticles have modest antimicrobial efficacy (more active against Gram‐positive bacteria) and are prized for chemical stability, whereas copper‐based compounds often exhibit stronger broad‐spectrum antimicrobial effects (attributable to Cu^2+^‐mediated oxidative stress and membrane disruption) [5, 6]. By forming a composite of Fe_2_O_3_/CuFe_2_O_4_ (copper ferrite spinel), the material can combine these features; magnetism from iron, redox activity from copper, and high surface area from nano structuring [7].

The prevalence of antibiotic‐resistant pathogens heightens the need for new antimicrobial agents. Foodborne and nosocomial infections caused by bacteria such as *Staphylococcus aureus* (Gram‐positive) and *Salmonella typhimurium* (Gram‐negative) pose serious public health risks, and yeasts like *Candida albicans* challenge immune‐compromised patients [8, 9]. Metal oxide nanocomposites have shown promise in this context, often by generating reactive oxygen species (ROS) or releasing metal ions that damage microbial cells [10, 11, 12]. Heterostructured composites can further enhance these mechanisms through interfacial charge transfer or dual‐mode action. For instance, Fenton‐type chemistry on iron oxide surfaces can produce •OH radicals from H_2_O_2_, and Cu^2+^ ions are known to disrupt enzyme function and membrane integrity. Thus, an Fe_2_O_3_/CuFe_2_O_4_ system is expected to be highly antimicrobial, with the possibility of tunable activity depending on phase composition and crystallinity [13, 14, 15].

Despite this promise, achieving controlled synthesis of Fe–Cu oxide composites with optimal phase purity and morphology remains challenging. Conventional one‐step calcination can lead to broad particle size distributions or unwanted phases [16, 17]. Here, we adopt a two‐stage calcination approach, systematically varying the calcination temperatures (e.g., 250°C and 300°C vs. 300°C and 400°C) to modulate crystallinity, phase proportion, and defect density. This method aims to optimize the Fe_2_O_3_ and CuFe_2_O_4_ interface. Comprehensive characterization (XRD, SEM, EDX) confirms phase identity and microstructure, while in vitro assays assess antimicrobial and antibiofilm efficacy against representative pathogens. Importantly, we also perform preliminary in vivo toxicological profiling (e.g., acute dosing in rodent models with histopathological and biochemical analysis) to evaluate biocompatibility, since successful antimicrobial agents must not harm host tissues. The present work builds upon recent developments in multifunctional nanostructures across biomedical and environmental domains. Beyond conventional formulations, eco‐friendly microwave synthesis utilizing agro‐industrial waste was successfully applied to fabricate antibacterial silver nanoparticles [18], whereas iron‐doped bismuth oxychloride nanorods demonstrated potent cytotoxic and apoptotic efficacy against osteosarcoma (MG‐63) cells [19]. Furthermore, bandgap modulation in iron‐doped bismuth oxychloride nanostructures achieved exceptional solar‐driven photocatalytic degradation of pharmaceutical pollutants [20]. In the context of Fe–Cu systems, Alnehia et al. reported a Fe_2_O_3_/CuFe_2_O_4_ composite with a large bandgap and selective activity against *S. aureus* [13], while others noted that CuFe_2_O_4_ imparts magnetic properties and enhances antibacterial effects under oxidative conditions [21]. However, systematic comparison of calcination protocols and evaluation of both Gram‐positive, Gram‐negative, and fungal targets along with in vivo safety assessment remain relatively unexplored.

Therefore, the primary objective of this study was to systematically evaluate the effect of two‐stage calcination parameters on the phase composition and structural properties of Fe_2_O_3_/CuFe_2_O_4_ nanocomposites (denoted BB1–BB4), and to investigate their broad‐spectrum antimicrobial/antibiofilm efficacy along with in vivo safety. Our findings demonstrate that the phase composition can be effectively tuned by the calcination sequence, with all composites exhibiting potent antimicrobial activity (showing sample‐dependent selectivity) and significant inhibition of pathogen biofilm formation. By integrating detailed structural characterization with in vitro and in vivo biological evaluations, this work offers new insights into optimizing synthesis conditions for developing safe and effective Fe–Cu oxide nanocomposites to address urgent public health challenges.

## 2 Experimental Procedure

### 2.1 Synthesis of Fe_2_O_3_/CuFe_2_O_4_ Nanocomposite

**SCHEME 1:**
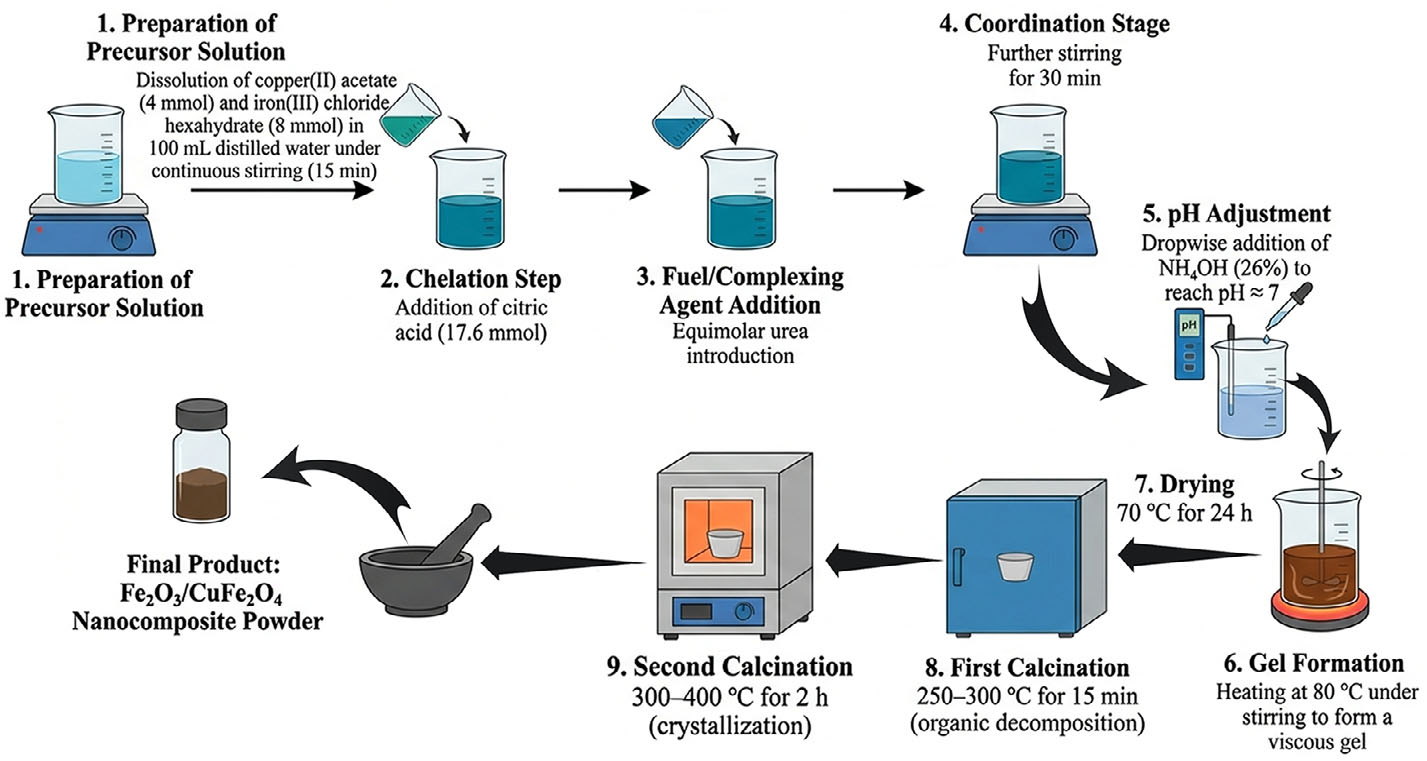
Schematic diagram for the Fe_2_O_3_/CuFe_2_O_4_ nanocomposite preparation.

The Fe_2_O_3_/CuFe_2_O_4_ nanocomposites were synthesized via a citric‐acid‐assisted sol–gel auto‐combustion route followed by a dual‐stage calcination process. As illustrated in Scheme 1, 4 mmol of copper (II) acetate and 8 mmol of F_2_Cl_3_.6H_2_O (iron (III) chloride hexahydrate) were dissolved in 100 mL of distilled water under continuous stirring for 15 min to obtain a clear and homogeneous solution containing Cu^2+^ and Fe^3+^ ions. Citric acid (17.6 mmol) was subsequently introduced as a chelating agent to coordinate the metal cations and regulate nucleation, followed by the addition of an equimolar amount of urea (17.6 mmol, equimolar to citric acid), which acts both as a complexing agent and as a fuel during thermal decomposition. The mixture was further stirred for 30 min to ensure complete coordination between the metal ions and organic ligands.

The Fe_2_O_3_/CuFe_2_O_4_ nanocomposites were synthesized via a citric‐acid‐assisted sol–gel auto‐combustion route followed by a dual‐stage calcination process. Initially, 4mmol of copper(II) acetate and 8mmol of $\mathrm{FeC}l_{3}\cdot 6H_{2}O$ (iron(III) chloride hexahydrate) were dissolved in 100mL of distilled water under continuous stirring for 15min to obtain a clear and homogeneous solution containing $Cu^{2+}$ and $Fe^{3+}$ ions. Citric acid (17.6mmol) was subsequently introduced as a chelating agent to coordinate the metal cations and regulate nucleation, followed by the addition of an equimolar amount of urea, which acts both as a complexing agent and as a fuel during thermal decomposition. The mixture was further stirred for 30min to ensure complete coordination between the metal ions and organic ligands.

The pH of the solution was adjusted to approximately 7 by the dropwise addition of 26% ammonium hydroxide (NH_4_OH). This adjustment facilitates the complete deprotonation of the carboxyl groups in citric acid, thereby maximizing its chelating affinity toward Cu^2+^ and Fe^3+^ ions, minimizing uncontrolled hydroxide precipitation, and promoting the formation of a homogeneous, stable metal–organic gel matrix. The solution was then heated at 80°C under continuous stirring to evaporate the solvent, leading to the formation of a viscous metal–organic gel. The resulting gel was dried at 70°C for 24 h to remove residual moisture and stabilize the precursor network.

The dried precursor underwent a two‐step calcination process at a controlled heating rate of 10°C/min. In the first stage, the sample was heated at 250°C–300°C for 15 min to decompose organic constituents and initiate phase evolution. In the second stage, calcination was carried out at 300°C–400°C for 2 h to promote crystallization. Table 1 illustrate sample nomenclature and dual‐stage calcination parameters for the synthesized Fe_2_O_3_/CuFe_2_O_4_ nanocomposites. Owing to the relatively moderate calcination temperatures, the final products consist of a mixed‐phase Fe_2_O_3_/CuFe_2_O_4_ nanocomposite, where Fe_2_O_3_ represents the dominant phase with a secondary CuFe_2_O_4_ spinel contribution.

**TABLE 1:** Sample nomenclature and dual‐stage calcination parameters for the synthesized Fe_2_O_3_/CuFe_2_O_4_ nanocomposites.

Sample code	Pre‐calcination	Second calcination
BB1	300°C for 15 min	400°C for 120 min
BB2	300°C for 15 min	300°C for 120 min
BB3	250°C for 15 min	400°C for 120 min
BB4	250°C for 15 min	300°C for 120 min

After natural cooling to room temperature, the obtained black powder was gently ground to achieve uniform particle distribution. This synthesis approach yields nanocomposites with controlled crystallinity and high surface area, which are advantageous for enhanced reactive oxygen species generation and improved interaction with microbial cell membranes due to the synergistic effect between Fe_2_O_3_ and CuFe_2_O_4_ phases.

The synthesized nanocomposites were characterized using x‐ray diffraction (XRD) for phase identification, scanning electron microscopy (SEM) for morphological analysis. Their antimicrobial activity was subsequently assessed using standard biological assays.

### 2.2 Characterization

#### 2.2.1 XRD

X‐ray diffraction (XRD) analysis was carried out using a Rigaku MiniFlex diffractometer (Rigaku Corporation, Tokyo, Japan) with Cu Kα radiation (*λ* = 1.5406 Å). The measurements were performed over a 2*θ* range of 10°–80° under standard operating conditions. The obtained diffraction patterns were used to identify the crystalline phases and evaluate the structural properties of the synthesized samples.

#### 2.2.2 SEM‐EDX

The surface morphology of the samples was examined using a scanning electron microscope (SEM, LEO EVO 40, Cambridge, UK). Prior to imaging, the samples were coated with a thin conductive layer using a BAL‐TEC SCD 050 sputter coater (Liechtenstein) to prevent surface charging. Elemental composition analysis was performed using an energy‐dispersive x‐ray spectroscopy (EDX) system (Bruker, 125 eV resolution, Berlin, Germany) attached to the SEM. The SEM‐EDX measurements were used to evaluate the morphological features and elemental composition of the synthesized samples.

### 2.3 Antibacterial and Antifungal Activity

#### 2.3.1 Bacterial and Fungal Strains

Reference and clinical pathogenic strains were used to assess antibacterial and anti‐biofilm activities, including Gram‐positive *S. aureus* (ATCC 25932), Gram‐negative *S. typhimurium* (ATCC 12048), and *C. albicans* (ATCC 10231) were maintained on nutrient agar slants for bacteria and Sabouraud dextrose agar for *C. albicans* at 4°C and freshly sub‐cultured before use.

#### 2.3.2 Samples Preparation

Nanoparticles were prepared at defined concentrations using sterile distilled water (SDW). All samples were sterilized by filtration through a 0.22 µm membrane filter [22].

#### 2.3.3 Bacterial Inoculum Preparation

Overnight cultures grown on nutrient agar for bacteria and Sabouraud dextrose agar for *C. albicans* at 37°C were suspended in sterile saline (0.85% NaCl) and adjusted to 0.5 McFarland standard (≈1 × 10^8^ CFU/mL) using a spectrophotometer at 600 nm.

#### 2.3.4 Disc Diffusion Assay (Kirby–Bauer Method)

To evaluate the growth‐inhiation profile of the NPs samples was evaluated accordance with CLSI guidelines using the disc diffusion method. Mueller–Hinton agar for bacteria and Sabouraud dextrose agar for *C. albicans* plates where a confluent bacterial layer was generated by swabbing the standardized inoculum across the agar surface. Sterile 6‐mm paper discs were impregnated with 10 µg/disc of the NPs, prepared as a suspension in sterile distilled water (SDW). An identical volume of the nanoparticle suspension was applied to each disc to maintain a consistent nanoparticle loading of 10 µg per disc. The impregnated discs were allowed to dry under sterile conditions before being placed on the inoculated agar surface using sterile forceps. The same nanoparticle loading, disc diameter, suspension volume, and drying procedure were maintained for all samples to improve inter‐disc reproducibility. Samples were placed on the agar surface at consistent intervals to prevent cross‐contamination. Commercial antibiotic discs tetracycline 10 µg (as a reference antibiotic control), fluconazole 64 µg (as a reference fungal control) were used as positive controls, incubated the plates at 37°C for 18–24 h, and the inhibition zones diameter was measured with a digital caliper [23].

#### 2.3.5 Minimum Inhibitory Concentration

To determine the MIC, apply the standardized broth microdilution procedure as per CLSI guidelines. In nutrient broth and Sabouraud dextrose broth for *C. albicans* 96‐well microtiter plates fold serial dilutions of each sample, each well received 100 µL of bacterial and fungal inoculum (≈5 × 10^5^ CFU/mL). Plates were incubated at 37°C for 18–20 h. MIC corresponded to the lowest concentration stopping visible growth, and evidence was achieved through spectrophotometric measurement at 600 nm [24].

#### 2.3.6 Minimum Bactericidal Concentration

MBC values determination procced by using 10 µL aliquots from MIC wells without visible growth were spot‐inoculated onto Mueller–Hinton agar for bacteria and Sabouraud dextrose broth for *C. albicans* plates and incubated at 37°C for 24 h. MBC determination was based on the lowest concentration causing ≥99.9% removal of viable bacterial cells qualified to the original inoculum [25].

#### 2.3.7 Preventive Biofilm Activity

The microtiter plate experiment was used to examine biofilm inhibition. Tryptic soy broth (TSB) mixed with 1% glucose was used to dilute bacterial colonies 1:100. After mixing 100 µL of the bacterial suspension and fungal suspension with 100 µL of the test sample at various concentrations in sterile 96‐well plates, the mixture was incubated for 24 h at 37°C. To remove planktonic cells, the wells were rinsed three times with phosphate‐buffered saline (PBS). Adherent biofilms were stained with 0.1% crystal violet for 30 min, washed, and air‐dried. The bound stain was solubilized with 95% ethanol, and absorbance was measured at 570 nm [26].

### 2.4 In Vivo Toxicological Evaluation

Twenty‐four (24) male albino rats (*Rattus norvegicus*) of about 250–300 g body weight were used. Males were preferred to avoid the physiological changes associated with the estrus cycle of females, which repeats every 4–6 days. The experiment was conducted between November 2025 and December 2025 in the animal house of the Medical Laboratory Science Department/College of Science/University of Raparin.

The animals were kept in plastic cages bedded with wooden chips, maintained at a controlled temperature of 22 ± 4°C, 12‐h light/dark cycles, and fed a standard diet and water ad libitum.

#### 2.4.1 Experimental Design

Rats were randomly divided into four groups of six animals each (*n* = 6).

*Group I Control*: Received standard rat diet and DW for 4 weeks.
*Group II Low dose NP (2.5 mg)*: Received NP (2.5 mg/kg BW, 3 days interval) by oral gavage for 4 weeks.
*Group III Medium dose NP (5 mg)*: Received NP (5 mg/kg BW, 3 days interval) by oral gavage for 4 weeks.
*Group IV High dose NP (10 mg)*: Received NP (10 mg/kg BW, 3 days interval) by oral gavage for 4 weeks.

Rats have free access to food and drinking water. The control group rats received an equivalent volume of distilled water through oral gavage for 4 weeks. BW was recorded regularly every 7 days to determine the administration doses during the study period. At the end of the study period (72 h after the last dose of the NP) animals were fasted overnight and then anesthetized by ketamine: xylazine mixture (90 mg/kg, i.p. and 10 mg/kg, i.p., respectively), blood samples were collected through cardiac puncture, put in gel tubes for 15 min, and finally centrifuged at 3000 rpm for 15 min. The obtained serum was analyzed for biochemical liver function test (LFT) parameters. Liver organs were also excised, washed with normal saline, and put in 10% formalin for histological examinations.

#### 2.4.2 Measurement of Serum Parameters

The levels of serum aspartate aminotransferase (AST), alanine aminotransferase (ALT), alkaline phosphatase (ALP), serum albumin (ALB), and total protein (TP) concentrations were estimated by using a full‐auto analyzer (cobas‐6000).

#### 2.4.3 Histological Study

The protocol used for the histological study depends on the literature procedure. Portions of the liver were fixed in 10% neutral formalin and further processed for pathological findings of hepatotoxicity.

### 2.5 Statistical Analysis

All experiments were performed in triplicate unless otherwise specified. Data are presented as mean ± standard deviation (SD) for in vitro assays and mean ± standard error (SE) for in vivo biological measurements. Statistical analyses were executed using GraphPad Prism software (Version 9.0, GraphPad Software, San Diego, CA, USA). Differences among experimental groups were evaluated using one‐way analysis of variance (ANOVA) followed by Duncan's post‐hoc test. A statistical probability level of <0.05 was considered statistically significant [27].

## 3 Results and Discussions

### 3.1 X‐Ray Diffraction (XRD) Analysis

**FIGURE 1:**
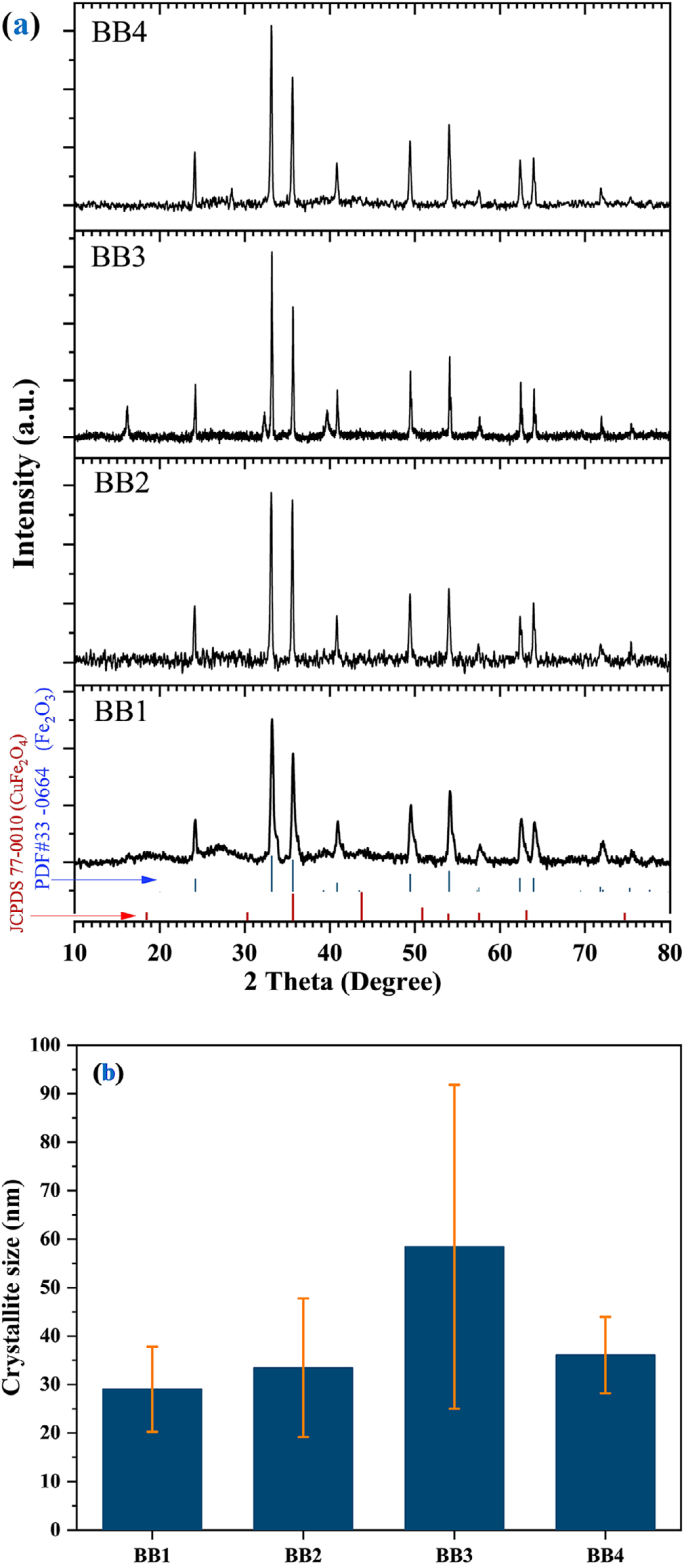
(a) X‐ray diffraction (XRD) patterns and (b) of BB1, BB2, BB3, and BB4 samples showing the crystalline phases of Fe_2_O_3_/CuFe_2_O_4_‐based structures.

The X‐ray diffraction (XRD) patterns of BB1, BB2, BB3, and BB4 samples are presented in Figure 1a. All samples exhibit sharp and well‐defined diffraction peaks, confirming the highly crystalline nature of the synthesized nanostructures. The major diffraction peaks are observed at approximately 2*θ* ≈ 24°, 33°, 36°, 41°, 50°, 54°, 58°, 62.5°, 64.5°, 72°, and 76°, which are characteristic of well‐crystallized oxide phases.

A detailed comparison with standard reference data reveals that these peaks are in excellent agreement with the rhombohedral α‐Fe_2_O_3_ phase (hematite, PDF#33‐0664) [28], indicating that Fe_2_O_3_ is the dominant crystalline phase in all samples. The high intensity and sharpness of these reflections further confirm the strong crystallinity and preferential formation of hematite under the employed synthesis and calcination conditions. In contrast, only a limited number of weak and partially overlapping peaks can be correlated with the spinel CuFe_2_O_4_ phase (JCPDS 77‐0010) [29]. The low intensity and poor resolution of these reflections suggest that CuFe_2_O_4_ is present as a secondary (minor) phase, likely due to incomplete solid‐state reaction and insufficient diffusion of Cu^2+^ ions into the spinel lattice at the relatively low calcination temperatures (300°C–400°C). This indicates that the system consists of a mixed‐phase Fe_2_O_3_/CuFe_2_O_4_ nanocomposite rather than a single‐phase spinel ferrite.

No noticeable peak shifts are observed among the samples, suggesting that the lattice parameters of the dominant Fe_2_O_3_ phase remain essentially unchanged with varying synthesis conditions. This behavior confirms the absence of significant lattice distortion or solid‐solution formation, further supporting the coexistence of two distinct crystalline phases rather than cation substitution within a single lattice. Moreover, no additional impurity peaks are detected beyond those associated with Fe_2_O_3_ and minor CuFe_2_O_4_ contributions, indicating that the synthesized nanocomposites are free from undesirable secondary phases. The variation in peak intensity among samples can be attributed to differences in crystallinity and relative phase proportions induced by changes in calcination temperature. Figure 1b illustrates the variation in crystallinity of the Fe_2_O_3_/CuFe_2_O_4_ nanocomposite samples (BB1, BB2, BB3, and BB4), which was quantitatively evaluated using the Scherrer equation:

(1) $$D=\frac{K\;\lambda }{\beta \cos\theta }\;$$

where 𝐷 is the average crystallite size, 𝐾 is the shape factor (typically ≈ 0.9), 𝜆 is the x‐ray wavelength, 𝛽 is the full width at half maximum (FWHM) of the diffraction peak (in radians), and 𝜃 is the Bragg diffraction angle. This method relates the crystallite size to the broadening of the diffraction peaks and provides an estimation of the average coherent domain size of the dominant crystalline phase, primarily Fe_2_O_3_ in the present system.

The crystallinity analysis was performed after background subtraction of the XRD patterns, followed by extraction of the FWHM values of the diffraction peaks associated with Fe_2_O_3_. The calculated crystallite sizes were approximately 29 ± 8, 34 ± 14, 58 ± 33, and 36 ± 8 nm for BB1, BB2, BB3, and BB4, respectively. These values indicate a general increase in crystallite size from BB1 to BB3, followed by a reduction in BB4. The observed increase in crystallite sizes up to BB3 is attributed to enhanced grain growth and improved crystallinity of the Fe_2_O_3_ phase at higher calcination temperatures (400°C), which facilitate atomic diffusion and crystal coalescence. This is consistent with the increased sharpness and intensity of Fe_2_O_3_ diffraction peaks observed in the XRD patterns. In contrast, the reduction in crystallite size for BB4 may be associated with the lower calcination temperature (300°C), which limits crystal growth and results in relatively smaller coherent domains.

It is important to note that the variation in crystallite size is primarily governed by thermal treatment conditions rather than compositional effects related to Cu incorporation, as CuFe_2_O_4_ exists only as a minor phase and does not significantly influence the overall crystallite size. Additionally, the relatively large standard deviations observed, particularly for BB3, suggest a broad crystallite size distribution, indicating structural heterogeneity that may arise from non‐uniform nucleation and growth during the sol–gel auto‐combustion process. Overall, the crystallite size analysis is consistent with the XRD results, confirming that the structural evolution of the nanocomposites is predominantly controlled by the crystallization behavior of the Fe_2_O_3_ phase under varying calcination conditions.

### 3.2 Surface Morphology and Elemental Composition Analysis (SEM–EDX)

**FIGURE 2:**
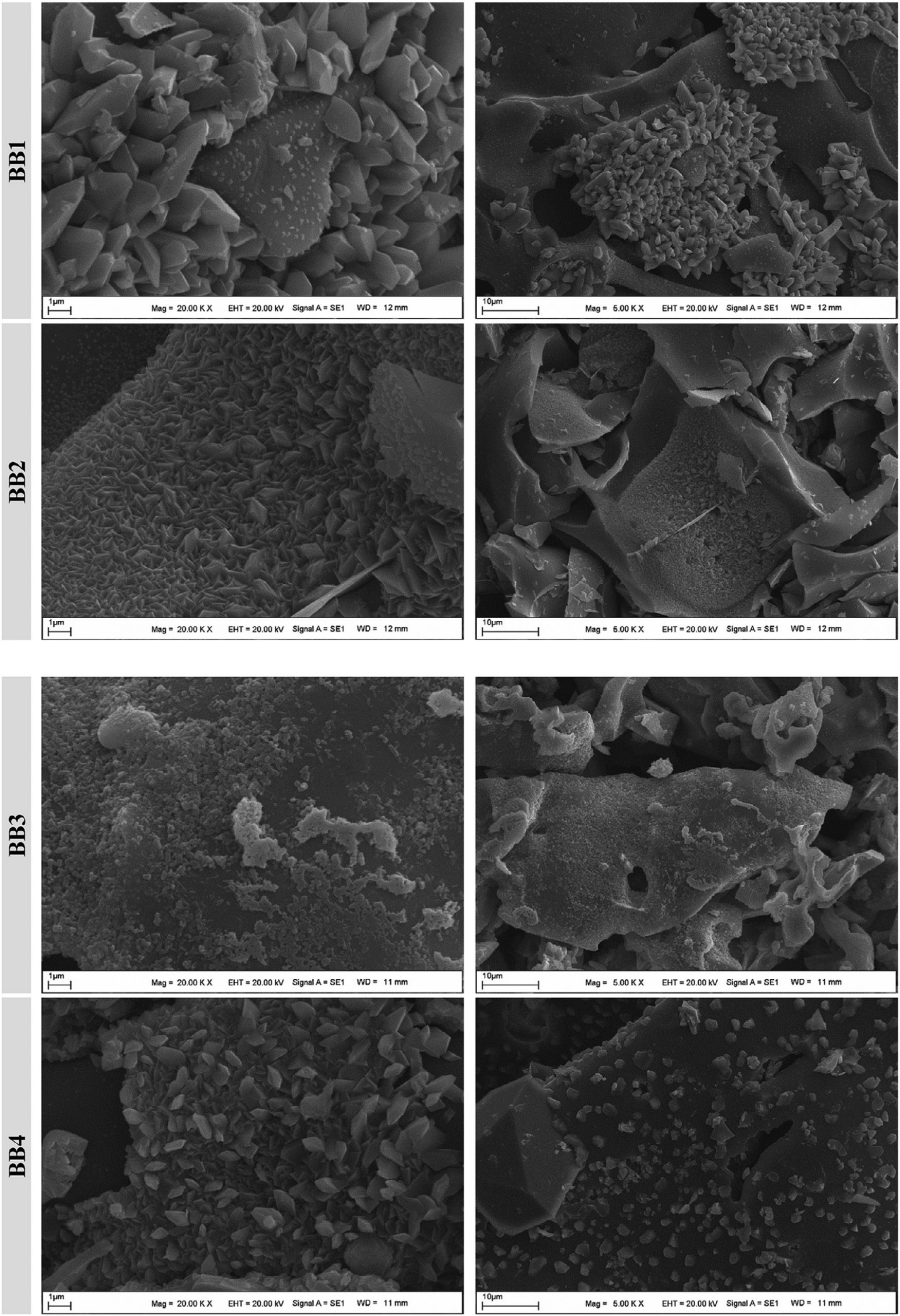
SEM micrographs of BB1, BB2, BB3, and BB4 samples showing morphological evolution with composition.

The surface morphology of the BB1, BB2, BB3, and BB4 samples was examined by scanning electron microscopy (SEM), and the corresponding images are presented in Figure 2. As clearly observed, all samples exhibit distinct morphological features depending on the composition, which is a common characteristic in spinel ferrite systems where synthesis conditions and composition strongly influence particle morphology [30, 31].

In the BB1 sample, a relatively well‐defined structure composed of plate‐like and irregularly shaped particles is observed. These particles appear to be densely packed, forming a compact morphology with locally clustered regions. This suggests that the initial composition promotes the formation of crystalline domains with relatively good structural integrity, consistent with SEM observations in ferrite nanoparticles showing compact and aggregated grain structures [32].

With increasing composition, particularly in the BB2 sample, a more uniform and densely distributed granular morphology is observed. The surface is covered with fine, closely packed crystalline features, indicating enhanced nucleation and growth processes. This behavior is commonly reported in ferrite systems, where optimized composition promotes uniform grain growth and improved particle distribution [33]. This observation is consistent with the XRD results, where an increase in crystallinity and crystallite size was detected.

In the BB3 sample, a noticeable change in morphology is observed, where the surface becomes more irregular and partially collapsed in certain regions. The presence of fragmented and less‐defined structures suggests that excessive composition may disrupt the structural organization, leading to a reduction in uniformity, which has been linked to defect formation and structural instability in doped ferrite systems [34].

For the BB4 sample, the morphology is characterized by the coexistence of larger particles and fine dispersed clusters. In addition, small spherical or near‐spherical particles are observed on the surface, indicating particle aggregation and secondary nucleation effects at higher composition levels. This behavior can be attributed to increased metal content, which promotes particle clustering and agglomeration, a commonly reported phenomenon in ferrite nanomaterials [35]. The SEM results demonstrate that the surface morphology is strongly influenced by compositional variations. The transition from compact and well‐defined structures to more heterogeneous and partially agglomerated morphologies is in good agreement with the XRD findings, confirming the relationship between composition, crystallinity, and structural evolution, as widely reported in recent ferrite studies [31].

In addition to the morphological observations, the elemental composition of the samples was analyzed by EDX, and the corresponding atomic percentages are presented in Table 2. The presence of Fe and Cu elements in all samples confirms the successful incorporation of the metal components into the structure. It is observed that the relative elemental ratios vary with composition, particularly the Fe content, which increases from BB2 to BB4, consistent with the structural changes observed in both XRD and SEM analyses.

**TABLE 2:** The atomic percentages of the elements, as obtained from the EDX analysis, for all synthesized nanoparticles (NPs).

Element	Atomic percentage (at.%)			
	BB1	BB2	BB3	BB4
Fe	12.42	7.61	12.05	16.17
Cu	14.07	6.69	17.17	10.49
Cl	11.61	13.37	13.95	14.53
N	19.4	24.66	17.81	20.44
C	27.6	31.02	22.7	22.97
O	14.91	16.65	16.32	15.39

Furthermore, the detection of C, N, and Cl atomic percentages (Table 2) reflects both analytical background features and surface‐bound precursor moieties. A significant contribution to the observed C and N levels arises from the conductive carbon tape substrate employed during SEM sample mounting, as well as the inherent semi‐quantitative nature and lower absorption corrections of EDX detectors toward light elements. Concurrently, the moderate dual‐stage calcination temperatures ($300^{\circ }C-400^{\circ }C$) facilitate the retention of trace surface‐coordinated organo‐chloride functionalities derived from the precursors ($\mathrm{FeC}l_{3}$, urea, and citric acid). These surface‐bound species are advantageous for biomedical applications, as residual surface charges and organic complexes enhance particle dispersibility in aqueous media and foster electrostatic adhesion to microbial cell membranes, thereby reinforcing biocidal interactions.

### 3.3 Antimicrobial and Antibiofilm Efficacy

#### 3.3.1 Agar Diffusion Antimicrobial Assay

**FIGURE 3:**
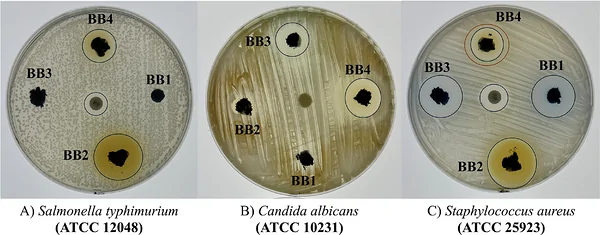
Antimicrobial activity of BB1, BB2, BB3, and BB4 synthesized nanoparticles against (A) *Salmonella*, (B) *Candida*, and (C) *Staphylococcus*.

Figure 3 and Table 3 of the study show the inhibition zones that samples BB1–BB4 made against *S. typhimurium* (a Gram‐negative bacteria), *S. aureus* (a Gram‐positive bacteria), and *C. albicans* (a fungus). The data demonstrates distinct the antimicrobial effect appears sample‐dependent rather than strictly concentration‐independent. and are significantly influenced by the sample. For *S. typhimurium*, sample BB2 created the biggest inhibition zone (22.0 ± 0.5 mm), which was much bigger than the tetracycline control's (11.0 ± 0.3 mm). BB4 also stopped *Salmonella* (15.0 ± 0.4 mm), but BB1 and BB3 had no effect (0 ± 0). This shows that only certain calcination conditions (BB2 and BB4) make particles that can get through or make reactive species that work against this Gram‐negative strain. All samples were active against *S. aureus*. BB2 had the biggest zone again (22.0 ± 0.3 mm), and BB1 (20.0 ± 0.2 mm), BB3 (20.0 ± 0.2 mm), and BB4 (15.0 ± 0.4 mm) also stopped *S. aureus*. The tetracycline control (14.0 ± 0.2 mm) is lower than these numbers. Gram‐positive bacteria, such as *S. aureus*, do not have the outer membrane that Gram‐negative bacteria do, which makes them more likely to be attacked by nanoparticles. Here, several nanocomposites successfully eradicated *S. aureus*, aligning with the established effectiveness of copper. For *C. albicans*, only BB3 and BB4 had antifungal properties, with zones of 14.0 ± 0.2 mm (BB3) and 18.0 ± 0.3 mm (BB4) being seen. BB1 and BB2 did not have an effect (0 ± 0). Fluconazole (positive control) produced no inhibition zone (0 mm), confirming that the *C. albicans* strain evaluated in this study is an azole‐resistant isolate. Notably, the ability of BB3 and BB4 to form distinct inhibition zones (14.0–18.0 mm) against this fluconazole‐resistant strain highlights the capacity of these nanocomposites to bypass conventional antifungal drug resistance mechanisms. The findings demonstrate that BB3/BB4 possess adequate active copper species (or surface defects) to impede yeast, in contrast to BB1/BB2, which lack such properties.

**TABLE 3:** Zone of inhibition (mm) of Fe_2_O_3_/CuFe_2_O_4_ nanocomposite against pathogenic microorganisms.

		Zone of inhibition (mm)					
Pathogenic microorganisms (PM)		BB1	BB2	BB3	BB4	Antibiotic tetracycline (TE, 10 µg/disc)	Antifungal fluconazole 64 µg/disc
*Salmonella typhimurium* ATCC 12048	Gram negative (−)	0 ± 0	22 ± 0.5^***^	0 ± 0	15 ± 0.4^**^	11 ± 0.3^**^	—
*Staphylococcus aureus* ATCC 25923	Gram positive (+)	20 ± 0.2^**^	22 ± 0.3^***^	20 ± 0.2^**^	15 ± 0.4^*^	14 ± 0.2^**^	—
*Candida albicans* ATCC 10231	Fungi	0 ±0	0 ±0	14 ± 0.2^**^	18 ± 0.3^***^	—	0 ± 0

*Note*: Data are expressed as mean ± SD (*n* = 3). One‐way ANOVA. Asterisks denote significance versus control (*p < 0.05, **p < 0.01, ***p < 0.001).

These agar‐diffusion results indicate that the effectiveness of antimicrobials depends on how they are made. For example, BB2 was the most effective against *Salmonella* and *S. aureus*, while BB4 was the most effective against *Candida*. The fact that BB2 worked much better than the antibiotic control for *Salmonella* and *S. aureus* suggests that it has a strong biocidal effect. A comparable investigation of a related Fe_2_O_3_–CuO–CuFe_2_O_4_ nanocomposite demonstrated efficacy solely against *S. aureus* (≈9 mm zone) and not against *Escherichia coli*, underscoring the significant impact of specific phases and preparation techniques on microbial selectivity [13, 36]. In our study, the binary Fe_2_O_3_/CuFe_2_O_4_ mixture exhibited efficacy against both Gram‐negative and Gram‐positive bacteria (and yeast), highlighting the significance of the copper ferrite phase.

#### 3.3.2 MIC and MBC Determinations

The minimum inhibitory concentration (MIC) and minimum bactericidal concentration (MBC) values, summarized in Table 4, complement the findings of the agar diffusion assay. All nanocomposites displayed strong antimicrobial efficacy, with MIC values occurring in the low tens of µg/mL. Against *S. typhimurium*, sample BB4 exhibited the highest potency, recording the lowest MIC and MBC values of 25 and 50 µg/mL, respectively. Samples BB1 and BB2 showed intermediate activity (MIC/MBC of 50/100 µg/mL), whereas BB3 was the least active formulation (100/200 µg/mL). A similar trend was observed for *S. aureus*, where BB4 consistently demonstrated the highest antibacterial performance (MIC/MBC of 25/50 µg/mL), followed by BB1 and BB3 (50/100 µg/mL), and BB2 (100/200 µg/mL). For *C. albicans*, samples BB2, BB3, and BB4 displayed equal antifungal potency with MIC/MBC values of 25/50 µg/mL, while BB1 exhibited slightly lower activity (50/100 µg/mL), confirming the antifungal efficacy shown in Table 3.

**TABLE 4:** Minimum inhibitory concentration (MIC) and minimum bactericidal concentration (MBC) of Fe_2_O_3_/CuFe_2_O_4_ nanocomposite against pathogenic microorganisms.

Agent	BB1		BB2		BB3		BB4	
Microorganism	MIC (µg/mL)	MBC (µg/mL)	MIC (µg/mL)	MBC (µg/mL)	MIC (µg/mL)	MBC (µg/mL)	MIC (µg/mL)	MBC (µg/mL)
*Salmonella typhimurium* ATCC 12048	50	100	50	100	100	200	25	50
*Staphylococcus aureus* ATCC 25923	50	100	100	200	50	100	25	50
*Candia albicans* ATCC 10231	50	100	25	50	25	50	25	50

The MIC and MBC determinations establish BB4 as the most potent formulation on a mass basis across both Gram‐positive and Gram‐negative bacterial strains. Notably, a distinct discrepancy is observed between the two testing modalities: BB2 produced the largest inhibition zones in the agar diffusion assay, whereas BB4 demonstrated superior efficacy in broth microdilution (lower MIC/MBC values). This phenomenon can be explained by the fundamental physical differences between solid and liquid assay environments. The agar diffusion assay is inherently mass‐transport‐limited, where zone size depends not only on biocidal reactivity but also on particle solubility, hydro‐dispersion, and the diffusion coefficient of released ions ($Cu^{2+}$) through the semi‐solid hydrogel matrix. The enhanced zone formed by BB2 suggests superior ionic mobility or lower steric/electrostatic interactions within the agar matrix. Conversely, the broth microdilution assay eliminates physical diffusion barriers through continuous suspension, directly evaluating mass‐normalized intrinsic cell interaction. The lower MIC/MBC values of BB4 reflect higher intrinsic antimicrobial reactivity, likely driven by optimized phase proportions ($Fe_{2}O_{3}/\mathrm{CuF}e_{2}O_{4}$), elevated surface defect density, and enhanced contact‐mediated reactive oxygen species (ROS) generation in liquid media [37, 38]. In summary, all synthesized nanocomposites exhibit remarkable broad‐spectrum antibacterial and antifungal activities at low working concentrations, performing comparably or superiorly to many previously reported metal oxide‐based antimicrobial systems [39, 40].

#### 3.3.3 Antibiofilm Activity

**FIGURE 4:**
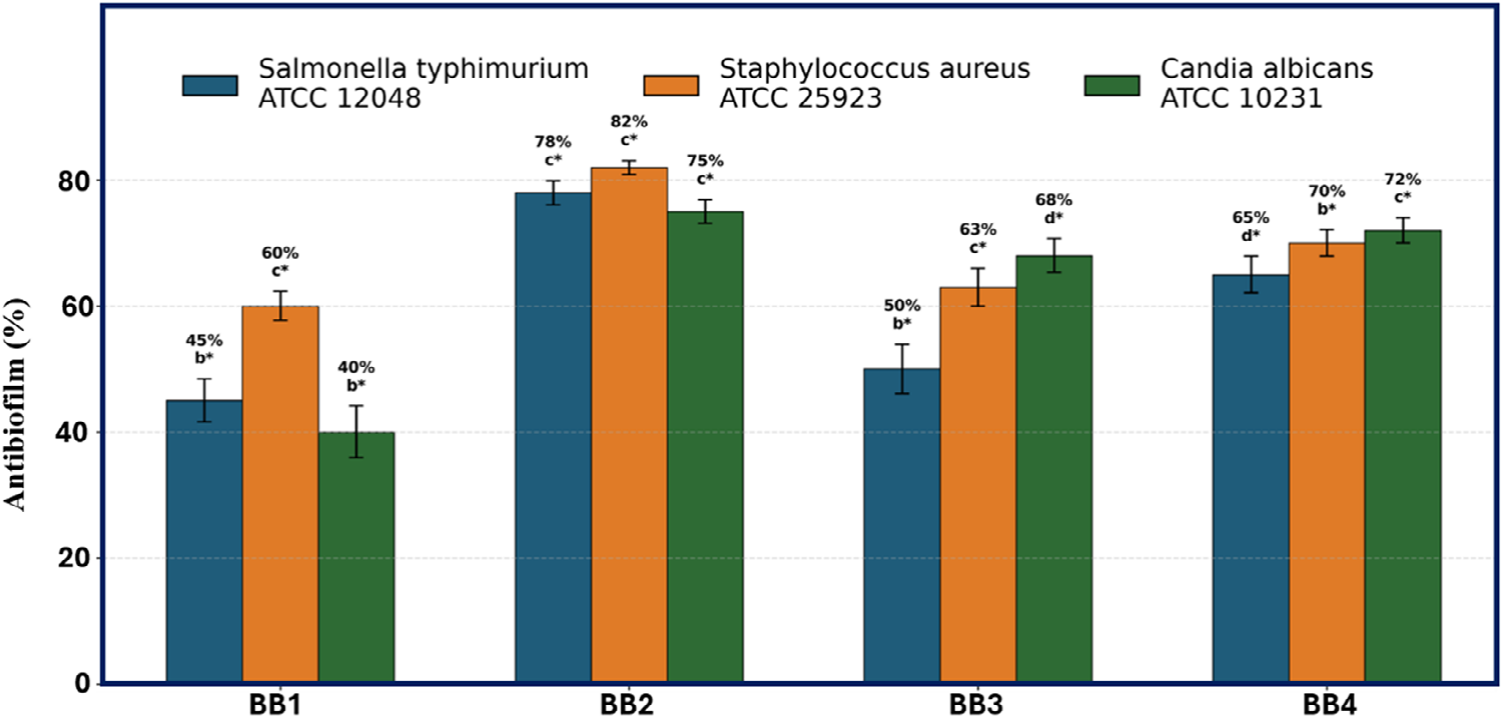
Antibiofilm activities of Fe_2_O_3_/CuFe_2_O_4_ nanocomposite against pathogenic microorganisms. Data are mean ± SD (*n* = 3 biological replicates). Statistical significance was determined by one‐way ANOVA followed by Duncan's multiple range test; within each organism, bars not sharing a letter differ significantly (*p* < 0.05); asterisks denote significance versus the untreated control (*p < 0.05).

Biofilm inhibition was quantified by measuring the absorbance of crystal‐violet‐stained biomass at OD_570_ in treated cultures (Figure 4 and Table 5). In tests involving *Salmonella*, *Staphylococcus*, and *Candida*, all samples significantly reduced biofilm formation compared to the untreated control. BB2 consistently exhibited the strongest antibiofilm activity among all the materials tested, leaving the least amount of residual biomass in all cases. It inhibited *Salmonella* by approximately 78%, *Staphylococcus* by 82%, and *Candida* by 75%. BB4 also demonstrated a significant effect on inhibition, while BB1 and BB3 had smaller but still notable effects. Overall, the results indicate that BB2 was the most effective sample, achieving about 70%–80% biofilm suppression. In contrast, the other samples generally exhibited more than 50% inhibition. These findings suggest that the Fe_2_O_3_/CuFe_2_O_4_ composite inhibits biofilm growth by disrupting cell adhesion and extracellular matrix formation, likely through oxidative and membrane‐damaging processes that contribute to its antimicrobial properties [14, 15, 41].

**TABLE 5:** Antibiofilm activities of Fe_2_O_3_/CuFe_2_O_4_ nanocomposite against pathogenic microorganisms.

Microorganism	Control OD_570_	BB1	BB2	BB3	BB4
*Salmonella typhimurium* ATCC 12048	1.20 ± 0.05^a^	0.66 ± 0.03^b^ ^*^	0.26 ± 0.02^c^ ^*^	0.60 ± 0.04^b^ ^*^	0.42 ± 0.03^d^ ^*^
*Staphylococcus aureus* ATCC 25923	1.10 ± 0.04^a^	0.44 ± 0.02^c^ ^*^	0.20 ± 0.01^c^ ^*^	0.41 ± 0.03^c^ ^*^	0.33 ± 0.02^b^ ^*^
*Candia albicans* ATCC 10231	1.3 ± 0.06^a^	0.78 ± 0.04^b^ ^*^	0.32 ± 0.02^c^ ^*^	0.42 ± 0.03^d^ ^*^	0.362 ± 0.02^c^ ^*^

*Note*: Data are demonstrated as mean ± SD (*n* = 3). Statistical significance was determined by one‐way ANOVA followed by for each parameter, means not followed by the same letter are significantly different by Duncan's multiple rang test (*p* > 0.05). Asterisks denote significance vs. control (*p < 0.05, **p < 0.01, ***p < 0.001).

#### 3.3.4 Mechanisms of Antimicrobial Action

The broad‐spectrum antimicrobial efficacy of the Fe_2_O_3_/CuFe_2_O_4_ composite is likely due to a number of synergistic mechanisms, with the generation of reactive oxygen species being the most important [42]. Fenton‐type reactions that happen on the surface of nanoparticles may change hydrogen peroxide into highly reactive hydroxyl radicals (•OH), which can hurt microbial DNA, proteins, and membrane lipids [43]. Although direct intracellular ROS quantification (e.g., via DCFH‐DA fluorescence assay or electron paramagnetic resonance) was not conducted in the present study, ROS generation is hypothesized as a key plausible pathway based on the surface defect chemistry of Fe–Cu oxide nanocomposites [15, 42]. The oxidative stress is probably made worse by copper redox cycling in the composite. Structural defects or heterojunctions may also make electron transfer easier and increase ROS production. In this situation, the Fe_2_O_3_/CuFe_2_O_4_ system could work well as a catalyst for long‐term oxidative damage to microbial cells [15].

The composite may also have antimicrobial properties by releasing metal ions and directly interacting with the cell surface, in addition to causing damage through ROS. In aqueous environments, there may be some leaching of Cu^2+^, Cu^+^, and Fe^3+^ ions. These ions are known to mess up the activity of enzymes, the integrity of membranes, and the balance of redox reactions in cells [44, 45]. The nanoparticles' positively charged surface may also cause them to be attracted to negatively charged microbial cell walls, which would bring them close to the cell envelope and focus oxidative and mechanical damage on the membrane. This kind of contact can cause the membrane to change shape, lose its charge, and break down lipids, which makes the cell even less likely to survive. These combined effects probably explain why both bacterial and fungal strains were so strongly inhibited [46, 47, 48].

In addition to planktonic cells, the nanocomposite seems to stop biofilm growth by stopping initial adhesion and breaking up the extracellular matrix that is needed for stable biofilm formation. The significant decrease in biofilm biomass recorded in the experimental data corroborates this interpretation and indicates that reactive oxygen species and released ions may compromise extracellular polymeric substances and disrupt signaling pathways critical for biofilm development [49, 50]. The composite's wider range of antimicrobial activity is probably due to the complementary effects of iron and copper. For example, Fe_2_O_3_ makes the catalyst more stable, and CuFe_2_O_4_ makes the biocidal effect stronger through copper‐related mechanisms. In general, the Fe_2_O_3_/CuFe_2_O_4_ nanocomposite has multiple ways to kill bacteria, making it a good choice for use in biomedical materials, antimicrobial coatings, and water treatment [51].

The observed variations in biocidal and antibiofilm potency among the synthesized samples (BB1–BB4) can be directly rationalized by quantitative phase estimation and crystallite size analysis derived from XRD data. Quantitative phase calculations reveal that the calcination regime significantly governs the relative yield of the active spinel CuFe_2_O_4_ phase within the dominant α‐Fe_2_O_3_ matrix. Specifically, sample BB2 exhibited the highest relative fraction of the $\mathrm{CuF}e_{2}O_{4}$ spinel phase (≈17.8 wt%) alongside α‐Fe_2_O_3_ (≈82.2 wt%) with an average crystallite size of 34±14nm. In contrast, BB1 (crystallite size 29 ± 8 nm), BB3 (58 ± 33 nm), and BB4 (36 ± 8 nm) contained lower relative spinel proportions of approximately 6.4, 1.4, and 1.2 wt%, respectively. The increased abundance of the copper ferrite phase in BB2 promotes a higher density of interfacial *p*–*n* heterojunctions, which facilitates efficient interfacial charge transfer, reduces electron–hole recombination, and enhances the exposure of redox‐active Cu^2+^ surface sites for ROS generation. Consequently, this phase distribution directly explains why BB2 displayed the most potent antibiofilm activity (78%−82% biomass reduction; $OD_{570}$= 0.20–0.26) and maximum zones of inhibition (22.0 mm for both *S. typhimurium* and *S. aureus*). Conversely, higher second‐stage calcination temperatures (400°C in BB3) promoted crystal growth (58 ± 33 nm) and reduced the active spinel fraction, thereby diminishing the overall biocidal efficacy.

Quantitative benchmarking against related Fe–Cu oxide systems further contextualize the antimicrobial performance observed in the present study. Bhushan et al. [52] reported α‐Fe_2_O_3_/CuO nanocomposites prepared by a wet‐chemical route, with MIC values of 600 µg mL^−1^ against *Bacillus subtilis*, *S. typhi*, and *E. coli*, and 750 µg mL^−^^1^ against *S. aureus* for their Cu‐rich FeC_4_ composition. More recently, an ultrasound‐assisted Fe_3_O_4_/CuO/chitosan nanocomposite exhibited MIC values of 300 µg mL^−^^1^ against *E. coli* and *B. subtilis* and 400 µg mL^−^^1^ against *S. cerevisiae* [53]. A CuFe_2_O_4_@SiO_2_‐polymer system showed comparatively higher MIC values in the range of 500–1000 µg mL^−^^1^ against Gram‐positive and Gram‐negative bacteria [54]. Conversely, more complex CuFe_2_O_4_‐containing hybrid architectures have achieved substantially lower MIC values; for example, a CuFe_2_O_4_‐containing MOF‐based composite exhibited MIC values of 50 µg mL^−^^1^ against *S. aureus* and *C. albicans* [55]. The MIC values obtained in this work nevertheless demonstrate that the Fe–Cu oxide system possesses competitive antimicrobial activity within the broader class of Fe–Cu‐based nanomaterials. A quantitative comparison of the MIC values, synthesis conditions, and target microorganisms of related Fe–Cu oxide systems is provided in Table 6.

**TABLE 6:** Quantitative comparison of antimicrobial performance of Fe–Cu oxide‐based nanomaterials reported in the literature.

Nanomaterial	Synthesis approach	Target organism	MIC	References
α‐Fe_2_O_3_/CuO Fe–Cu oxide nanocomposite (FeC4)	Wet‐chemical synthesis; Cu‐precursor composition varied	*B. subtilis*	60 mg dL^−^^1^	[52]
			(600 µg mL^−^^1^)	
α‐Fe_2_O_3_/CuO Fe–Cu oxide nanocomposite (FeC4)	Wet‐chemical synthesis; Cu‐precursor composition varied	*S. aureus*	75 mg dL^−^^1^	
			(750 µg mL^−^^1^)	
α‐Fe_2_O_3_/CuO Fe–Cu oxide nanocomposite (FeC4)	Wet‐chemical synthesis; Cu‐precursor composition varied	*S. typhi*	60 mg dL^−^^1^	
			(600 µg mL^−^^1^)	
α‐Fe_2_O_3_/CuO Fe–Cu oxide nanocomposite (FeC4)	Wet‐chemical synthesis; Cu‐precursor composition varied	*E. coli*	60 mg dL^−^^1^	
			(600 µg mL^−^^1^)	
Fe_3_O_4_/CuO/chitosan nanocomposite	Ultrasound‐assisted/green synthesis	*E. coli*	0.30 mg mL^−^^1^	[53]
			(300 µg mL^−^^1^)	
Fe_3_O_4_/CuO/chitosan nanocomposite	Ultrasound‐assisted/green synthesis	*B. subtilis*	0.30 mg mL^−^^1^	
			(300 µg mL^−^^1^)	
Fe_3_O_4_/CuO/chitosan nanocomposite	Ultrasound‐assisted/green synthesis	*S. cerevisiae*	0.40 mg mL^−^^1^	
			(400 µg mL^−^^1^)	
CuFe_2_O_4_@SiO_2_‐polymer	Polymer‐assisted composite synthesis	*S. aureus*, *E. coli*, *P. aeruginosa*	500–1000 µg mL^−^^1^	[54]
CuFe_2_O_4_/MoS_2_/MIL‐101(Co)‐based composite	Hydrothermal/in‐situ composite synthesis	*S. aureus*	50 µg mL^−^^1^	[55]
CuFe_2_O_4_/MoS_2_/MIL‐101(Co)‐based composite	Hydrothermal/in‐situ composite synthesis	*C. albicans*	50 µg mL^−^^1^	

### 3.4 In Vivo Toxicity and Hepatotoxicity

**FIGURE 5:**
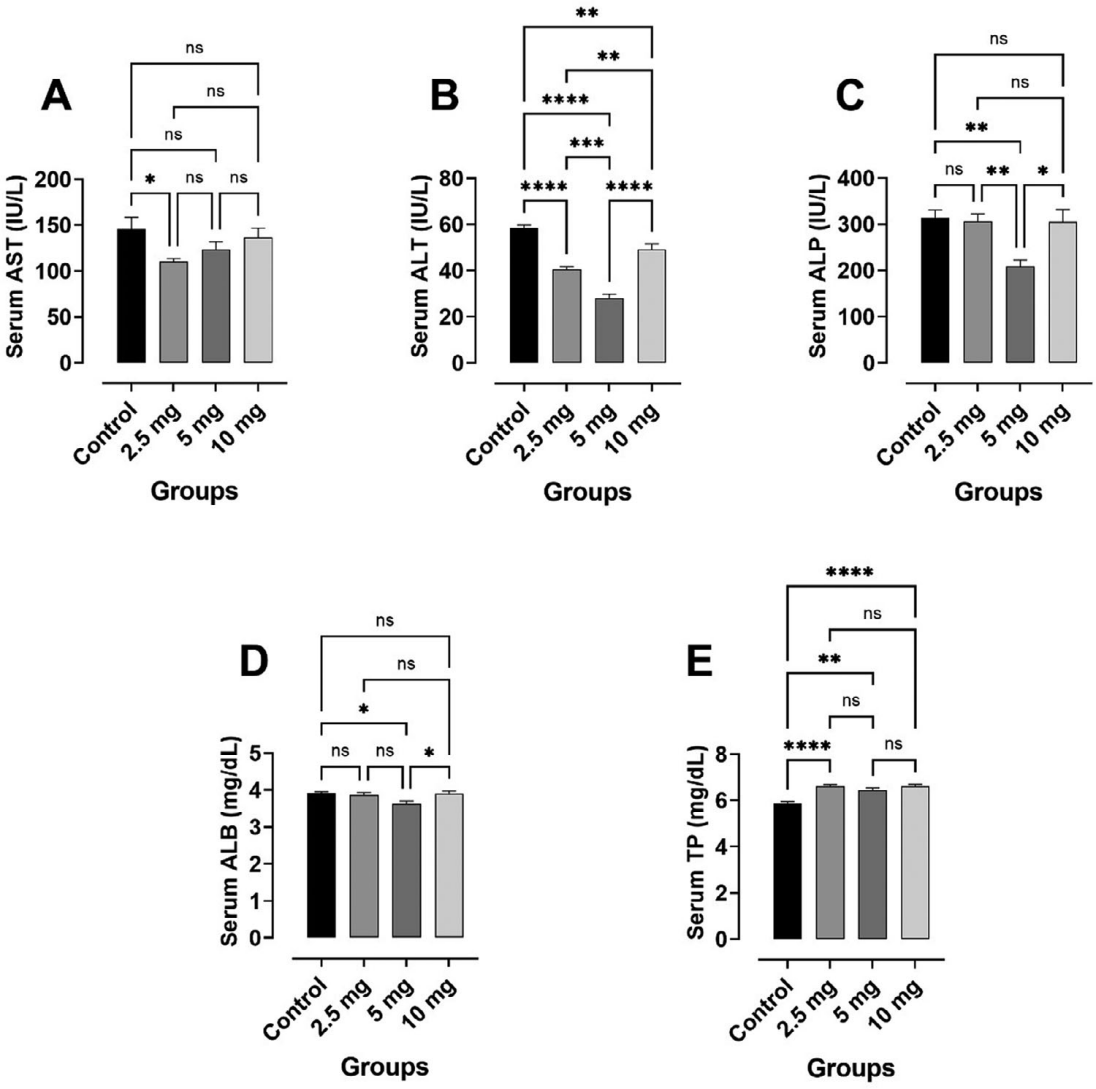
The effect of different doses of NP administration on serum liver function parameters. (A) AST, (B) ALT, (C) ALP, (D) albumin, and (E) TP in experimental rats. Data are presented as mean ± SD. Statistical differences between groups are indicated as: *p<0.05, **p<0.01, ***p<0.001, ****p<0.0001; ns: non‐significant (p>0.05).

The findings demonstrated variable hepatic responses to Fe_2_O_3_/CuFe_2_O_4_ nanocomposites (see Figure 5), with no clear dose‐dependent trend; however, several biochemical markers indicate mild hepatocellular and metabolic alterations. A modest increase in AST at 2.5 mg/kg (*p* < 0.05), accompanied by a pronounced reduction in ALT levels across all treated groups (*p* < 0.001 to *p* < 0.0001), suggests an initial and limited hepatocellular membrane perturbation at lower doses, followed by possible adaptive stabilization or antioxidant‐mediated protection at higher concentrations [56]. Similarly, the significant reduction in ALP activity at 2.5 and 5 mg/kg (*p* < 0.01), followed by a slight increase at 10 mg/kg (*p* < 0.05), may reflect dose‐dependent modulation of biliary function or metabolic enzyme activity, consistent with reports indicating that metal oxide nanoparticles can induce transient oxidative or cholestatic stress.

The observed decrease in serum albumin at 5 and 10 mg/kg (*p* < 0.05), together with the reduction in total protein at 2.5 and 5 mg/kg (*p* < 0.01 to *p* < 0.0001), indicates a mild and reversible impairment in hepatic synthetic function. The partial recovery of total protein levels at the highest dose further supports the presence of physiological adaptation mechanisms [57, 58]. Overall, these results are in agreement with previous studies demonstrating that metal oxide nanocomposites can temporarily disturb liver enzyme homeostasis through mechanisms involving reactive oxygen species generation, mitochondrial dysfunction, and lipid peroxidation, while compensatory cellular responses may attenuate these effects under higher or prolonged exposure conditions [59, 60].

The biochemical parameters of liver function, including AST, ALT, ALP, serum albumin, and total protein, provide important insights into hepatocellular stress and liver integrity following exposure to Fe_2_O_3_/CuFe_2_O_4_ nanocomposites. The data indicate that AST levels increased at lower doses, suggesting early hepatocellular membrane disturbance and leakage of intracellular enzymes into circulation. In contrast, ALT levels showed a non‐monotonic decrease across all treated groups. Beyond cellular adaptation, this non‐linear response may reflect toxicological hormesis (i.e., a biphasic overcompensation activating endogenous defenses) or potential assay interference, where residual serum nanocomposites interfere with optical enzyme detection. At the highest dose (10 mg/kg), partial normalization of these markers was observed, likely due to activation of endogenous antioxidant defense systems or cellular adaptation processes [56, 58].

A moderate increase in ALP activity at higher doses (5 and 10 mg/kg) may indicate mild biliary dysfunction or metabolic stress, which is commonly associated with oxidative and inflammatory responses in hepatic tissue [61]. Additionally, the reduction in serum albumin and total protein levels suggests a transient impairment of hepatic biosynthetic activity under nanoparticle exposure. These observations are consistent with previous reports indicating that metal oxide nanomaterials can induce oxidative stress‐mediated hepatotoxicity via ROS generation, mitochondrial damage, and lipid peroxidation pathways [59, 60]. Altogether, while these dose‐dependent responses reflect subtle metabolic stress, the lack of severe hepatic injury supports the moderate biocompatibility of the nanocomposite, though further in vitro cytotoxicity assays remain valuable for mechanistic clarity.

Pure iron oxide (Fe_2_O_3_) and copper ferrite (CuFe_2_O_4_) nanoparticles exhibit distinct performance profiles, with the latter generally showing higher antimicrobial potency at significantly lower concentrations. Specifically, pure Fe_2_O_3_ produces a substantial bacterial inhibition zone of approximately 33 mm against *E. coli* at a concentration of 20 mg/mL [62], whereas green‐synthesized CuFe_2_O_4_ achieves a 17 mm zone at just 0.1 mg/ml [63]. In toxicological studies, Fe_2_O_3_ demonstrates selective toxicity with an IC_50_ of 12.87 µg/mL against MCF‐7 cancer cells 40.24 µg/mL and against normal Vero cells [62]. In comparison, CuFe_2_O_4_ shows IC_50_ values between 40 µg/mL and 100 µg/mL for various cancer lines [64] and exhibits greater safety for normal cells, such as PC12, with an IC_50_ of 225.01 µg/ml [65]. While has a well‐documented oral No‐Observed‐Adverse‐Effect Level (NOAEL) of 500 mg/kg in rats [66], safety constraints for complex ferrites typically involve tighter clinical windows to avoid adverse physiological effects [64].

## 4 Conclusions

Synthesis of heterostructured Fe_2_O_3_/CuFe_2_O_4_ nanocomposite via a regulated two‐stage calcination technique. It was discovered to possess potent antibacterial effects. Structural investigation verified that α‐Fe_2_O_3_ is the predominant crystalline phase, with small inclusions of CuFe_2_O_4_, and crystallite sizes demonstrated a direct relationship with the calcination process. The investigated samples exhibiting the most potent biocidal effects were BB2 (300°C and 300°C) and BB4 (250°C and 300°C). BB2 exhibited the largest zones of inhibition against *S. typhimurium* and *S. aureus*, but BB4 and BB3 showed superior efficacy against *C. albicans*. MIC/MBC data corroborated these findings. For instance, BB4 had the lowest inhibitory doses against bacteria, while all Cu‐rich samples showed activity against the fungus. All nanocomposites significantly inhibited biofilm formation (often exceeding 50% inhibition), with BB2 demonstrating a reduction of about 70%–80% across several strains. The remarkable efficacy of BB2 and BB4 underscores the importance of calcination conditions in modulating active sites and surface chemistry. An increased calcination temperature (400°C) likely promoted crystal formation and improved crystallinity, whereas lower‐temperature phases preserved defects or active copper sites. These supplementary ingredients contributed to a broad spectrum of activity against both Gram‐negative and Gram‐positive microorganisms. Crucially, preliminary in vivo toxicity investigations revealed that the improved nanocomposite did not induce substantial organ damage or inflammation, suggesting an acceptable safety profile for further development. The Fe_2_O_3_/CuFe_2_O_4_ nanocomposite exhibits a distinctive combination of potent antibacterial properties and minimal acute toxicity. This research provides a foundation for utilizing mixed Fe–Cu oxide compounds as novel antibacterial agents. Future research should explore complex mechanisms (e.g., ROS production routes), long‐term biocompatibility, and practical applications, including coatings or wound dressings. The present findings highlight a substantial need for innovative antibacterials and antifungals, advancing the science toward nanoscale approaches to infection management. Further studies involving in vitro cytocompatibility assays using normal mammalian cells, particularly fibroblast or keratinocyte models, are warranted to establish the cellular safety profile of the nanocomposite before biomedical application.

## Author Contributions

**K. A. Babakr** and **I. N. Qader**: supervision, project administration, conceptualization, review. **Asmaa Sayed Ahmed** and **Chawan Hazhar Razaq**: biological activity and writing. **Sleman Yousif Omar** and **Zana Hassan Ibrahim**: cytotoxicity and synthesis NPs. **Riyadh Zainadin Mawlood** and **Mustafa Ersin Pekdemir**: writing and visualization.

## Ethics Statement

All animal procedures and experimental protocols conducted in this study were reviewed and approved by the Institutional Review Board (IRB) Committee at the University of Raparin (Approval No. 85, Ref: 2866/28‐5‐2023).

## Conflicts of Interest

The authors declare no conflicts of interest.

## Declaration of Generative AI and AI‐Assisted Technologies in the Writing Process

During the preparation of this work, the authors utilized GPTEdit to enhance the English language and improve the clarity of the manuscript. After using this tool, the authors reviewed and edited the content as necessary and took full responsibility for the publication's content.

## Data Availability

The data that support the findings of this study are available from the corresponding author upon reasonable request.
